# FRID-PI: a machine learning model for diagnosing fracture-related infections based on ^18^F-FDG PET/CT and inflammatory markers

**DOI:** 10.3389/fmed.2025.1534988

**Published:** 2025-03-26

**Authors:** Mei Yang, Quanhui Tan, Tingting Li, Jie Chen, Weiwei Hu, Yi Zhang, Xiaohua Chen, Jiangfeng Wang, Chentian Shen, Zhenghao Tang

**Affiliations:** ^1^Department of Infectious Diseases, Shanghai Sixth People's Hospital Affiliated to Shanghai Jiao Tong University School of Medicine, Shanghai, China; ^**2**^Department of Nuclear Medicine, Shanghai Sixth People's Hospital Affiliated to Shanghai Jiao Tong University School of Medicine, Shanghai, China

**Keywords:** ^18^F-FDG PET/CT, laboratory biomarkers, fracture-related infection, nomogram, model

## Abstract

**Purpose:**

The diagnosis of fracture-related infection (FRI) especially patients presenting without clinical confirmatory criteria in clinical settings poses challenges with potentially serious consequences if misdiagnosed. This study aimed to construct and evaluate a novel diagnostic nomogram based on ^18^F-fluorodeoxyglucose positron emission tomography /computed tomography (^18^F-FDG PET/CT) and laboratory biomarkers for FRI by machine learning.

**Methods:**

A total of 552 eligible patients recruited from a single institution between January 2021 and December 2022 were randomly divided into a training (60%) and a validation (40%) cohort. In the training cohort, the Least Absolute Shrinkage and Selection Operator (LASSO) regression model analysis and multivariate Cox regression analysis were utilized to identify predictive factors for FRI. The performance of the model was assessed using the area under the Receiver Operating Characteristic (ROC) curve (AUC), calibration curves, and decision curve analysis in both training and validation cohorts.

**Results:**

A nomogram model (named FRID-PE) based on the maximum standardized uptake value (SUV_max_) from ^18^F-FDG PET/CT imaging, Systemic Immune-Inflammation Index (SII), Interleukin - 6 and erythrocyte sedimentation rate (ESR) were generated, yielding an AUC of 0.823 [95% confidence interval (CI), 0.778–0.868] in the training test and 0.811 (95% CI, 0.753–0.869) in the validation cohort for the diagnosis of FRI. Furthermore, the calibration curves and decision curve analysis proved the potential clinical utility of this model. An online webserver was built based on the proposed nomogram for convenient clinical use.

**Conclusion:**

This study introduces a novel model (FRID - PI) based on SUV_max_ and inflammatory markers, such as SII, IL - 6, and ESR, for diagnosing FRI. Our model, which exhibits good diagnostic performance, holds promise for future clinical applications.

**Clinical relevance statement:**

The study aims to construct and evaluate a novel diagnostic model based on ^18^F-fluorodeoxyglucose positron emission tomography /computed tomography (^18^F-FDG PET/CT) and laboratory biomarkers for fracture-related infection (FRI).

## Highlights

- The diagnosis of fracture-related infection (FRI) especially patients presenting without clinical confirmatory criteria is difficult.
- ^18^F-FDG PET/CT and Machine Learning is increasingly used in infectious diseases.
- The model based on ^18^F-FDG PET/CT and inflammatory markers contributes to the diagnostic process as well as to further decision-making.

## Introduction

Fracture-related infection (FRI) represents a formidable challenge in modern trauma surgery (1), with significant global implications. The 2019 Global Burden of Diseases, Injuries, and Risk Factors Study (GBD) estimated an annual incidence of 178 million new bone fractures, totaling 2,296 cases per 100,000 population (2). The prevalence of FRI is expected to rise in the coming decade due to factors such as increased life expectancy, a growing incidence of osteoporotic and age-related fragility fractures, and a rise in surgical fracture repair procedures globally (3). FRIs invariably necessitate long-term follow-up and high pain tolerance, multiple surgical interventions, extended antimicrobial therapy, the achievement of bony union, and the restoration of diminished bone or joint functionality and can even escalate to the point of necessitating amputation or posing life-threatening risks (4). Hence, early identification and diagnosis are crucial for timely decision-making regarding surgical intervention or alternative therapeutic strategies. Notably, unlike prosthetic joint infections (PJI), which have more established diagnosis and treatment guidelines, FRIs present unique challenges due to associated bone and soft tissue damage and the option of implant removal after fracture healing.

In clinical practice, orthopedic surgeons typically classify FRIs into two primary groups: the first includes patients with clear clinical indicators of infection, such as fistula, sinus, wound breakdown, or purulent drainage from the wound. The second group, lacking clear clinical confirmatory criteria (5), poses a diagnostic challenge, where laboratory findings serve as suggestive yet relatively ambiguous criteria (6).

In this context, the use of imaging tools becomes invaluable for surgeons, aiding in the selection of the optimal therapeutic approach. These tools not only help identify signs of infection but also play a crucial role in assessing implant stability and bone regeneration. However, each imaging technique has its limitations. For instance, plain radiography falls short in evaluating soft tissue and exhibits limited sensitivity and specificity for FRI diagnosis (7). Ultrasound faces constraints in bone assessment due to metallic implants. Computed tomography (CT) struggles with soft tissue contrast and the identification of early signs of infection like bone marrow edema (8). Magnetic resonance imaging (MRI) is relatively ineffective in post-traumatic conditions and fails to distinguish reactive bone marrow edema (9). Besides, both CT and MRI are impeded by metallic implants.

In recent years, several nuclear imaging techniques have been introduced to enhance infection diagnosis. Systematic reviews have indicated high diagnostic accuracy for nuclear imaging (10), but these techniques generally have low anatomical resolution. Three-phase bone scintigraphy, despite widespread use, has limited specificity and diagnostic value in violated bone (<1–2 years after trauma/fracture/osteosynthesis, <5 years after arthroplasty), making it unsuitable as a primary diagnostic tool for FRI (7, 11, 12). Gallium scans are time-consuming, requiring approximately 48–72 h. White blood cell scintigraphy (WBCS) is also time-consuming, and there is no standardized protocol (9).

In contrast, ^18^F-fluorodeoxyglucose positron emission tomography/computed tomography (^18^F-FDG PET/CT), though expensive, offers a relatively rapid turnaround time (60 min post-tracer injection) and is gaining popularity in infection/inflammatory diseases. The imaging interpretation is presented in two forms: visual image analysis, providing ^18^F-FDG uptake patterns and anatomical position, and semiquantitative analysis using standardized uptake value (SUV), total lesion glycolysis (TLG), etc. Multiple studies have validated its superior sensitivity compared to other nuclear medicine techniques in chronic osteomyelitis of the extremities (7, 8, 11). Furthermore, systematic reviews have confirmed its high diagnostic accuracy in detecting spinal infections (13). Nonetheless, both ^18^F-FDG PET/CT and laboratory findings exhibit a limited ability to isolate FRI, and research or guidelines about FRI diagnosis and treatment remain relatively scarce (14). Nevertheless, the potential of ^18^F-FDG PET/CT in detecting infections appears promising.

Neither laboratory findings nor imaging alone can diagnose FRI. It is widely thought that the diagnostic value could be enhanced by combining the two in diagnosing FRI. Hyunkwang et al. (15) combined SUV_max_ (maximum standardized uptake value) and blood inflammatory markers, building a model to assess treatment response in pyogenic vertebral osteomyelitis. The results demonstrated that the combined performance of SUV_max_ and blood inflammatory markers was superior to the blood inflammatory markers alone.

Nomograms are frequently used tools for prognostic estimations in both oncology and general medicine. They can amalgamate determinant variables, making them increasingly prevalent in tumor prognosis (16, 17). However, their utility extends to diagnostics as well (18). Given their characteristics as an integrated model, they significantly contribute to the advancement of personalized medicine (19).

In our study, we aimed to develop a novel nomogram based on ^18^F-FDG PET/CT and laboratory findings to refine early FRI diagnosis ability. Considering that the diagnosis of FRI in patients with clear clinical confirmatory criteria is relatively straightforward and does not require comprehensive tests, the primary aim of our study was to provide insight into the diagnosis and management of a subpopulation of FRI patients presenting without clinical confirmatory criteria.

## Methods

We conducted an extensive search for patients who underwent ^18^F-FDG PET/CT scans and other laboratory examinations due to post-fracture pain at Shanghai Sixth People’s Hospital between January 2021 and December 2022.

The inclusion criteria were as follows:

- underwent surgery for a fracture;
- suspect infection for prolonged postoperative pain;
- the time interval between the latest surgery and ^18^F-FDG PET/CT scan was more than 3 months;
- the time interval between the ^18^F-FDG PET/CT scan and blood tests, following surgery or open biopsy was <1 month;
- no antibiotic pretreatment within 1 week before ^18^F-FDG PET/CT scan;
- complete clinical data;
- complete histopathologic or microbiologic cultivation results.

The exclusion criteria were as follows:

- pathological fractures due to malignant bone tumors;
- poor image quality;
- other causes of bone infections (e.g., PJI, diabetic feet, spondylodiscitis, and hematogenous osteomyelitis);
- non-extremity bones (e.g., skull and maxilla);
- patients with other comorbidities (e.g., tumors, autoimmune diseases, multiple myeloma, and other system inflammations) that might affect serum biomarker levels.

**Figure 1:**
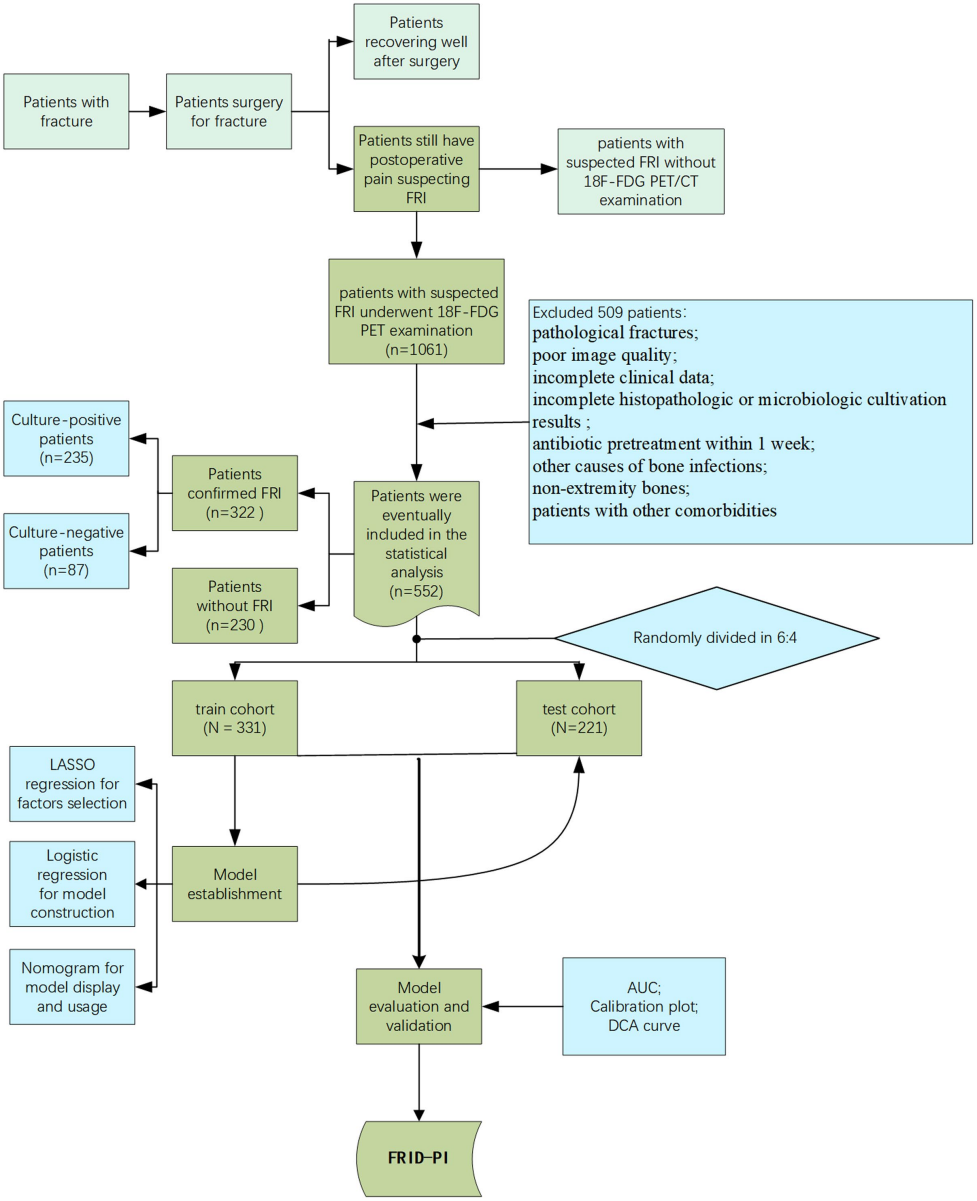
The participant recruitment process is illustrated in the flowchart.

The participant recruitment process is illustrated in the flowchart in Figure 1.

### Collection of basic clinical and laboratory data

Patients’ clinical data collection is collected and organized through the electronic medical record system, including:

- Basic information: gender, age, number of days of hospitalization, number of surgeries;
- Blood biochemical indexes: blood routine and related indexes [white blood cell count (WBC), red blood cell count (RBC), platelet count, percentage of neutrophils, absolute value of lymphocytes (ALC), absolute value of monocytes (AMC), absolute value of neutrophils, mean platelet volume (MPV), mean platelet distribution width (PDW), platelet corpuscle (PCT), neutrophil-monocyte ratio, neutrophil-to-lymphocyte ratio (NLR), monocyte-to-lymphocyte ratio (NLR), and platelet pressure (PCT), neutrophil-to-monocyte ratio (NMR), neutrophil-to-lymphocyte ratio (NLR), monocyte-to-lymphocyte ratio (MLR), platelet-to-lymphocyte ratio (PLR), platelet count-to-mean platelet volume (P/V), platelet count-to-mean platelet distribution width (PADW), platelet count-to-platelet hematocrit (PTP), systemic inflammatory immunity index (SII), systemic inflammatory immunity index (SII), and systemic inflammatory diseases (SII), Systemic Inflammatory Response Index (SIRI)];
- Coagulation-related markers [Prothrombin Time (PT), International Normalized Ratio (INR), D-dimer, Prothrombin Time, Activated Partial Thromboplastin Time (APTT), Fibrinogen (FIB), Thromboplastin Time (TT), Fibrinogen Degradation Product (FDP), and Anticoagulase III (AT-III)];
- Nutrition-related markers [albumin, albumin-to-globulin ratio (AGR), Prognostic Nutritional Index (PNI)];
- Inflammation-related indicators [C-reactive protein (CRP), calcitonin (PCT), interleukin-6, erythrocyte sedimentation rate (ESR)];

Calculation of relevant indicators:

PNI = 10 × serum albumin (g/dL) + 5 × Lymphocyte count (*10^9/L).

PLR = Platelet count (*10^9/L)/Lymphocyte count (*10^9/L).

NLR = Neutrophil count (*10^9/L) / Lymphocyte count (*10^9/L).

MLR = Monocyte count (*10^9/L) / Lymphocyte count (*10^9/L).

SII = Platelet count (*10^9/L) × Neutrophil count (*10^9/L) / Lymphocyte count (*10^9/L).

SIRI = Neutrophil count (*10^9/L) × Monocyte count (*10^9/L) / Lymphocyte count (*10^9/L).

The assessment of FRI adhered to the diagnostic criteria established by a consensus reached by an international expert group in 2019 (6):

- Fistula, sinus, or wound breakdown;
- Purulent drainage from the wound or presence of pus during surgery;
- Phenotypically indistinguishable pathogens identified by culture from at least two separate deep tissue/implant specimens;
- Presence of microorganisms in deep tissue taken during an operative intervention, as confirmed by histopathological examination.

Other suggestive criteria were not confirmed diagnoses in this study. Histopathological features indicating FRI included necrotic bone, fibrosis, bony repair, or organisms on histological specimens; polymorphonuclear (PMN) cells >5 per high-power field (20) and direct identification of a microorganism from deep-tissue specimens using specific staining techniques (6). A microbiological culture was considered positive if any relevant organism had grown based on the judgment of a microbiologist. Those not meeting the above diagnostic criteria were classified as the non-FRI group.

### ^18^F-FDG PET/CT imaging

Patients were instructed to fast for at least 6 h before the injection of ^18^F-FDG. Blood glucose level was measured before injection and ^18^F-FDG was administered at glucose levels < 11.1 mmol/L. ^18^F-FDG PET/CT scanning was performed after an i.v. injection of 3-4 MBq/Kg ^18^F-FDG, followed by an 1-h uptake phase. No intravenous contrast agent was administered. ^18^F-FDG PET/CT scans were performed using a dedicated GE Discovery PET/CT scanner including 64 slice CT scanners with a dedicated PET (BGO plus crystal). ^18^F-FDG images were acquired for 2.5 min at each bed position from the skull base to the superior mediastinum with patients’ arms along the chest and from the neck to the mid-thigh with patients’ arms above the head. No specific breathing instructions were given. The CT scan was obtained from the orbitomeatal line and progressed to the mid-thigh with the use of a standardized protocol involving 120 kV, 80 mA, and a slice thickness of 3.75 mm. Attenuation correction of PET images was performed using attenuation data from CT and image reconstruction was done using a standard reconstruction algorithm with ordered subset expectation maximization (OSEM). Image fusion was performed using coordinate-based fusion software and subsequently reviewed at a workstation (Xeleris) that provided multi-planar reformatted images and displayed PET, CT, and PET/CT fusion images.

### Statistical analysis

While preoperative laboratory examinations, such as complete blood count (CBC) and coagulation function, play a crucial role, they tend to be more abundant in modeling. In the training cohort, the Minimum-Redundancy Maximum-Relevance (mRMR) algorithm (21) and the Least Absolute Shrinkage and Selection Operator (LASSO) method (22) were employed to identify the most relevant and robust features. The mRMR algorithm aims to identify features highly correlated with the diagnosis of FRI while minimizing correlations with other features, thus mitigating model overfitting. The LASSO method, designed for high-dimensional data regression, was utilized to select features with non-zero coefficients.

All statistical analyses were carried out using R Studio software (version 4.3.0). LASSO regression was implemented using the “glmnet” package. Statistical significance was set at *p* < 0.05 (two-sided). Differences between the training and validation cohorts were evaluated using the independent *t*-test or Mann–Whitney *U* test, depending on the data distribution. Chi-squared testing was employed to assess the significance of differences between categorical variables. Model performance was assessed with a focus on discrimination and calibration.

### Model construction and evaluation

#### Discrimination

To assess the diagnostic performance of the model, we generated Receiver Operating Characteristic (ROC) curves. The optimal cutoff values of the biomarkers, calculated from the training cohort, were then applied in the validation cohort. A bar chart was generated to visually represent the discrimination performance. DeLong testing was employed to compare the Area Under ROC Curves (AUC) between the training and validation cohorts.

#### Calibration

Calibration curves were generated for both the training and validation cohorts to investigate the agreement between the observed and predicted outcomes of the model. The goodness of fit was assessed using Hosmer-Lemeshow testing, and *p*-values exceeding 0.05 were indicative of well-calibrated models.

#### Decision curve analysis (DCA)

In the validation cohort, DCA was conducted to evaluate the clinical application of the model. DCA is a method for assessing the net benefits of predictive models (23).

## Results

### Clinical characteristics

A total of 552 patients meeting the predetermined inclusion and exclusion criteria were identified from the hospital information system databases. The cohort, which consisted of 427 males and 125 females, had an average age of around 50 years. Of these, 322 (58.4%) were diagnosed with FRI based on the specified diagnostic criteria. Table 1 outlines the basic characteristics of the two groups. Indicators such as SUV_max_, NLR, SII, WBC, CRP, the percentage of neutrophils, neutrophils, IL-6, and ESR were significantly higher in the group of patients diagnosed with FRI, while indicators such as albumin, AGR, MPV, and PDW were significantly lower in the FRI patient group. No significant differences in gender, age, MLR, RBC, ALC, platelet hematocrit, TT, or AT-III were observed at baseline (*p* > 0.05). As expected, the FRI group had a longer length of hospital stay and a higher number of surgeries (*p* < 0.05).

**Table 1:** Baseline characteristics for patients in non-FRI and FRI groups.

	FRI	non-FRI	*P-*value^**2**^
	*N* = 322^**1**^	*N* = 230^**1**^	
Gender (%)			0.986
Male	249 (77.3%)	178 (77.4%)	
Female	73 (22.7%)	52 (22.6%)	
Age (years)	51 (36, 62)	50 (36, 58)	0.358
Days of hospitalization	11.0 (8.0, 16.0)	9.0 (7.3, 11.8)	<0.001
Number of surgeries	2.00 (1.00, 3.00)	2.00 (1.00, 3.00)	<0.001
SUV_max_	6.30 (4.80, 7.88)	3.70 (2.90, 5.10)	<0.001
PNI	417 (377, 449)	425 (399, 457)	0.002
MLR	0.27 (0.20, 0.38)	0.25 (0.20, 0.33)	0.058
NLR	2.32 (1.71, 3.30)	1.91 (1.41, 2.60)	<0.001
PLR	131 (101, 179)	121 (93, 160)	0.022
SII	8,216 (5,614, 11,892)	6,765 (4,933, 9,700)	<0.001
SIRI	1.11 (0.75, 1.93)	0.89 (0.60, 1.27)	<0.001
Albumin (g/L)	40.7 (37.0, 44.0)	41.8 (39.0, 45.0)	0.001
AGR	1.30 (1.10, 1.43)	1.40 (1.30, 1.60)	<0.001
WBC (*10^9/L)	7.10 (5.80, 8.70)	6.15 (5.00, 7.28)	<0.001
CRP (mg/L)	7 (3, 16)	6 (1, 8)	<0.001
RBC (*10^12/L)	4.76 (4.27, 5.15)	4.81 (4.39, 5.13)	0.43
Platelet count (*10^9/L)	240 (192, 315)	228 (182, 279)	0.005
Neutrophil percentage (%)	63 (56, 69)	58 (52, 64)	<0.001
ALC (*10^9/L)	1.80 (1.50, 2.30)	1.80 (1.50, 2.30)	0.888
AMC (*10^9/L)	0.50 (0.40, 0.60)	0.50 (0.40, 0.60)	0.017
ANC (*10^9/L)	4.50 (3.40, 5.70)	3.60 (2.70, 4.30)	<0.001
MPV (fl)	10.00 (9.40, 11.08)	10.45 (9.80, 11.20)	0.002
PDW (%)	11.30 (10.00, 13.08)	11.80 (10.70, 13.58)	0.005
Platelet hematocrit (%)	0.25 (0.20, 0.31)	0.23 (0.20, 0.29)	0.057
Platelet count/mean platelet volume ratio (P/V)	24 (18, 32)	22 (17, 28)	0.001
Platelet count/mean platelet distribution width	22 (15, 29)	19 (14, 25)	<0.001
Platelet count/platelet hematocrit	996 (904, 1,066)	957 (884, 1,022)	<0.001
Platelet count/albumin	5.94 (4.72, 7.64)	5.27 (4.30, 6.65)	<0.001
PCT (ng/ml)	0.050 (0.025, 0.061)	0.034 (0.020, 0.041)	<0.001
IL-6 (pg/ml)	10 (3, 15)	5 (2, 7)	<0.001
ESR (mm/h)	32 (15, 39)	18 (9, 21)	<0.001
D-dimer (mg/L FEU)	0.59 (0.32, 1.10)	0.42 (0.24, 0.75)	<0.001
PT (s)	11.90 (11.30, 12.60)	11.70 (11.10, 12.20)	0.002
INR	1.04 (0.98, 1.10)	1.02 (0.97, 1.06)	<0.001
APTT (s)	27.90 (26.23, 29.90)	27.30 (25.90, 29.10)	0.004
FIB (g/L)	2.97 (2.51, 3.77)	2.61 (2.35, 3.01)	<0.001
TT (s)	17.00 (16.30, 17.70)	17.00 (16.40, 17.80)	0.467
FDP (mg/L)	2.00 (2.00, 3.50)	2.00 (2.00, 2.58)	0.002
AT-III (%)	92 (85, 97)	91 (86, 99)	0.633

^1^*n* (%); median (interquartile range). ^2^chi-square independence test; Wilcoxon rank sum test; *p* < 0.05 were considered statistically significant. SUV_max_, Maximum standardized uptake value; PNI, Prognostic Nutritional Index; MLR, Monocyte-to-Lymphocyte Ratio; NLR, Neutrophil-to-Lymphocyte Ratio; PLR, Platelet-to-Lymphocyte Ratio; SII, Systemic Immune-Inflammation Index; SIRI, System Inflammation Response Index; AGR, albumin to globulin ratio; WBC, white blood cell count; CRP, C-reactive protein; RBC, red blood cell count; ALC, absolute lymphocyte count; AMC, absolute monocyte count; ANC, absolute neutrophil count; MPV, mean platelet volume; PDW, mean platelet distribution width; PCT, procalcitonin; Interleukin-6; ESR, erythrocyte sedimentation rate; PT, prothrombin time; INR, international normalized ratio; APTT, activated partial thromboplastin time; FIB, fibrinoge; TT, thrombin time; FDP, Fibrinogen degradation products; AT-III, antithrombin III.

Among the 552 patients, 322 patients were diagnosed with FRI, and 195 patients had positive culture results. The positive bacterial identification rate in microbiological cultures was 60.6% (*n* = 195), with methicillin-sensitive *Staphylococcus aureus* being the predominant causative bacterium (29.4%, *n* = 69). Further details on bacteriology are provided in Table 2.

**Table 2:** Culture results of microorganisms.

Identification of causative microorganisms	No. of patients
*Staphylococcus aureus*	103
MSSA	69
MRSA	34
Polymicrobial infection	35
*Pseudomonas aeruginosa*	17
*Klebsiella pneumoniae*	10
Aerobacter cloacae	8
*Staphylococcus epidermidis*	7
*Enterococcus faecalis*	7
*E. coli Escherichia Coli*	6
*Streptococcus agalactiae*	5
*Lactobacillus rhamnosus*	4
*Candida albicans*	3
*Mycobacterium tuberculosis*	2
Acinetobacter Baumanii	2
*Staphylococcus aureus*	2
*Pseudomonas aeruginosa*	2
*Streptococcus pyogenes*	2
*Clostridium tetani*	2
*Morganella morganii*	1
*Streptococcus pneumoniae*	1
*Corynebacterium diphtheriae*	1
*Staphylococcus aureus*	1
*Pseudomonas aeruginosa*	1
*Achromobacter xylosoxidans*	1
*Bacillus cereus*	1
*Clostridium botulinum*	1
*Salmonella enterica*	1
*Escherichia coli*	1
*Staphylococcus aureus*	1
*Streptococcus constellatus*	1
*Staphylococcus aureus*	1
Bidirectional Prevotella	1
*Mycobacterium avium* Pennei	1
Klebsiella pnenmoniae	1
*Serratia marcescens*	1
Candida pseudohyphae	1

MSSA, methicillin-sensitive *Staphylococcus aureus*; MRSA, methicillin-resistant *Staphylococcus aureus*.

All 552 patients were randomly assigned to either the training cohort (60%, *N* = 331) or the validation cohort (40%, *n* = 221) using R Studio software. No significant variations in clinical characteristics were observed between the two cohorts (*P* > 0.05), as shown in Table 3

**Table 3:** Clinical characteristics of patients.

	Training cohort	Validation cohort	*P*-value^2^
	*N* = 331^1^	*N* = 221^1^	
Gender (%)			0.685
Male	258 (77.9%)	169 (76.5%)	
Female	73 (22.1%)	52 (23.5%)	
Age (years)	50 (36, 62)	50 (39, 61)	0.93
Days of hospitalization	9.0 (8.0, 13.0)	10.0 (8.0, 15.0)	0.006
Number of surgeries	2.00 (1.00, 3.00)	2.00 (1.00, 3.00)	0.379
SUV_max_	5.00 (3.50, 7.05)	5.30 (3.70, 7.10)	0.354
PNI	426 (392, 454)	415 (380, 450)	0.075
MLR	0.25 (0.20, 0.36)	0.26 (0.20, 0.36)	0.726
NLR	2.11 (1.65, 3.07)	2.13 (1.54, 2.89)	0.421
PLR	129 (98, 175)	124 (100, 169)	0.542
SII	7,597 (5,262, 11,170)	7,232 (5,250, 10,765)	0.392
SIRI	1.03 (0.67, 1.67)	1.02 (0.72, 1.57)	0.958
Albumin (g/L)	41.6 (38.1, 44.5)	40.3 (37.0, 44.0)	0.062
AGR	1.40 (1.20, 1.50)	1.30 (1.20, 1.50)	0.065
WBC (*10^9/L)	6.40 (5.45, 7.90)	6.80 (5.60, 8.20)	0.204
CRP (mg/L)	7 (1, 10)	7 (1, 9)	0.711
RBC (*10^12/L)	4.79 (4.37, 5.17)	4.73 (4.30, 5.13)	0.477
Platelet count (*10^9/L)	232 (188, 304)	240 (183, 293)	0.686
Neutrophil percentage (%)	61 (55, 67)	60 (54, 67)	0.338
ALC (*10^9/L)	1.80 (1.40, 2.30)	1.90 (1.50, 2.30)	0.356
AMC (*10^9/L)	0.50 (0.40, 0.60)	0.50 (0.40, 0.60)	0.172
ANC (*10^9/L)	3.90 (3.10, 5.00)	3.90 (3.20, 5.30)	0.494
MPV (fl)	10.20 (9.55, 11.05)	10.30 (9.60, 11.20)	0.323
PDW (%)	11.30 (10.25, 13.35)	11.80 (10.40, 13.20)	0.447
Platelet hematocrit (%)	0.24 (0.20, 0.29)	0.24 (0.20, 0.30)	0.412
Platelet count/mean platelet volume ratio (P/V)	23 (17, 30)	24 (17, 29)	0.839
Platelet count/mean platelet distribution width	21 (14, 28)	21 (15, 27)	0.891
Platelet count/platelet hematocrit	986 (904, 1,052)	970 (891, 1,045)	0.168
Platelet count/albumin	5.50 (4.62, 7.21)	5.90 (4.48, 7.55)	0.354
PCT (ng/ml)	0.036 (0.020, 0.061)	0.041 (0.025, 0.061)	0.074
IL-6 (pg/ml)	7 (3, 15)	7 (3, 15)	0.643
ESR (mm/h)	20 (11, 35)	20 (11, 35)	0.381
D-dimer (mg/L FEU)	0.46 (0.28, 0.94)	0.54 (0.29, 0.99)	0.212
PT(s)	11.80 (11.20, 12.40)	11.80 (11.30, 12.40)	0.616
INR	1.03 (0.97, 1.08)	1.03 (0.98, 1.08)	0.648
APTT(s)	27.60 (25.95, 29.70)	27.70 (25.90, 29.40)	0.747
FIB (g/L)	2.73 (2.44, 3.32)	2.87 (2.46, 3.58)	0.114
TT(s)	17.00 (16.30, 17.80)	17.00 (16.30, 17.70)	0.966
FDP (mg/L)	2.00 (2.00, 3.00)	2.00 (2.00, 3.30)	0.203
AT-III (%)	91 (86, 97)	92 (84, 98)	0.783

^1^*n* (%); median (interquartile range). ^2^Pearson’s chi-square test; Wilcoxon rank sum test; *p* < 0.05 were considered statistically significant. SUV_max_, Maximum standardized uptake value; PNI, Prognostic Nutritional Index; MLR, Monocyte-to-Lymphocyte Ratio; NLR, Neutrophil-to-Lymphocyte Ratio; PLR, Platelet-to-Lymphocyte Ratio; SII, Systemic Immune-Inflammation Index; SIRI, System Inflammation Response Index; AGR, albumin to globulin ratio; WBC, white blood cell count; CRP, C-reactive protein; RBC, red blood cell count; ALC, absolute lymphocyte count; AMC, absolute monocyte count; ANC, absolute neutrophil count; MPV, mean platelet volume; PDW, mean platelet distribution width; PCT, procalcitonin; Interleukin-6; ESR, erythrocyte sedimentation rate; PT, prothrombin time; INR, international normalized ratio; APTT, activated partial thromboplastin time; FIB, fibrinogen; TT, thrombin time; FDP, Fibrinogen degradation products; AT-III, antithrombin III.

### Model construction and evaluation

**Figure 2:**
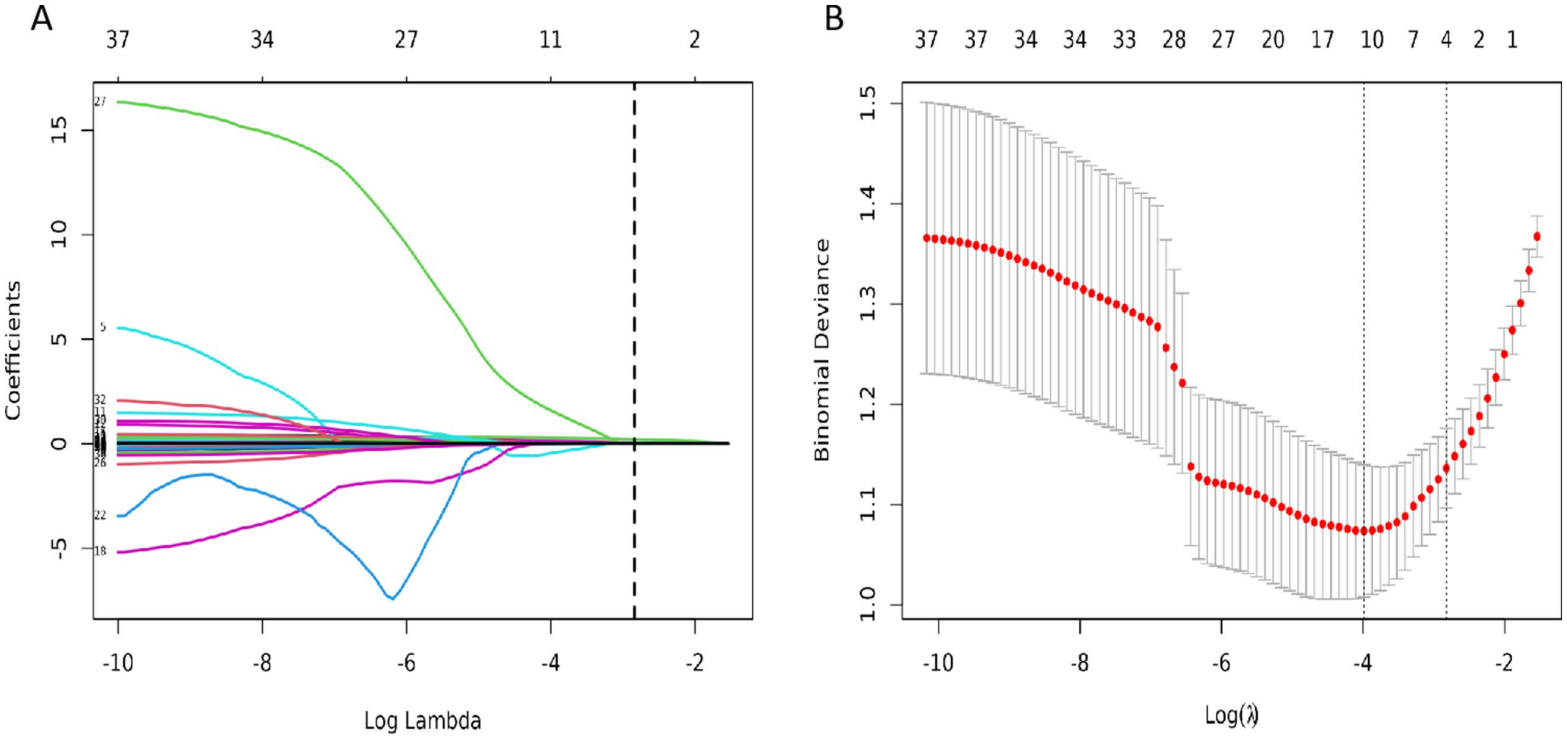
**(A)** The 35 variables in the training cohort of infection were screened by LASSO regression. The optimal Lambda parameter was selected used by 10-fold cross validation. The Lambda value was taken as the optimal value of the model when the cross validation error was minimum, and the number of variables corresponding to the non-zero regression coefficient was counted at this time; **(B)** LASSO coefficients for infection-related factors in the training cohort.

**Figure 3:**
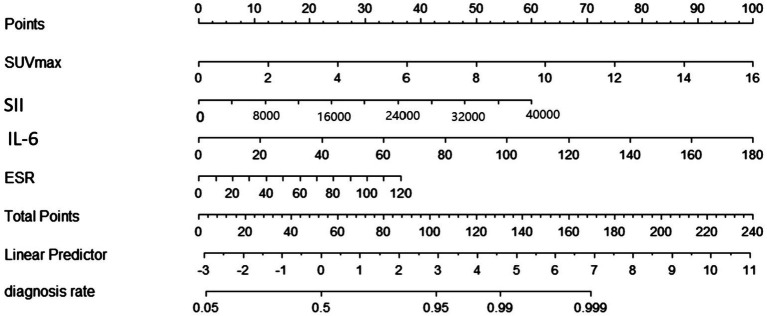
Nomogram for diagnose FRI in the training group. FRI = fracture-related infection.

In the training set, four significant predictors of FRI were identified from among the 35 laboratory biomarkers and SUV_max_ (Figures 2A,B). These predictors, including SUV_max_, SII, ESR, and IL - 6, were initially screened using Lasso regression. Logistic multifactorial regression analysis revealed that SUV_max_ (*p* < 0.001, 95% CI: 1.22–1.59), SII (*p* = 0.026, 95% CI: 1.02–1.38), IL - 6 (*p* = 0.022, 95% CI: 1.00–1.07), and ESR (*p* = 0.014, 95% CI: 1.00–1.04) were independent predictors for FRI (Table 4). Consequently, the final FRID - PI nomogram is depicted in Figure 3.

**Table 4:** Multivariate logistic analysis of potential diagnose factors identified by LASSO regression in the training cohort.

Parameters	OR^1^	95% CI^1^	*P*-value
SUV_max_	1.39	1.22, 1.59	<0.001
SII	1.19	1.02, 1.38	0.026
IL-6 (pg/ml)	1.03	1.00, 1.07	0.022
ESR (mm/h)	1.02	1.00, 1.04	0.014

^1OR, Odds Ratio; 95% CI:, 95% confidence interval; SUVmax, Maximum standardized uptake value; SII, Systemic Immune-Inflammation Index; ESR, erythrocyte sedimentation rate; IL-6, leukocytes-6.^

### Performance of the model

The discriminative ability of the diagnostic model was assessed using ROC curves. In the training set, the AUC was 0.823 [95% confidence interval (CI), 0.778–0.868], and the AUC in the validation was 0.811 (95% CI, 0.753–0.869), indicating a moderate discriminative ability.

**Figure 4:**
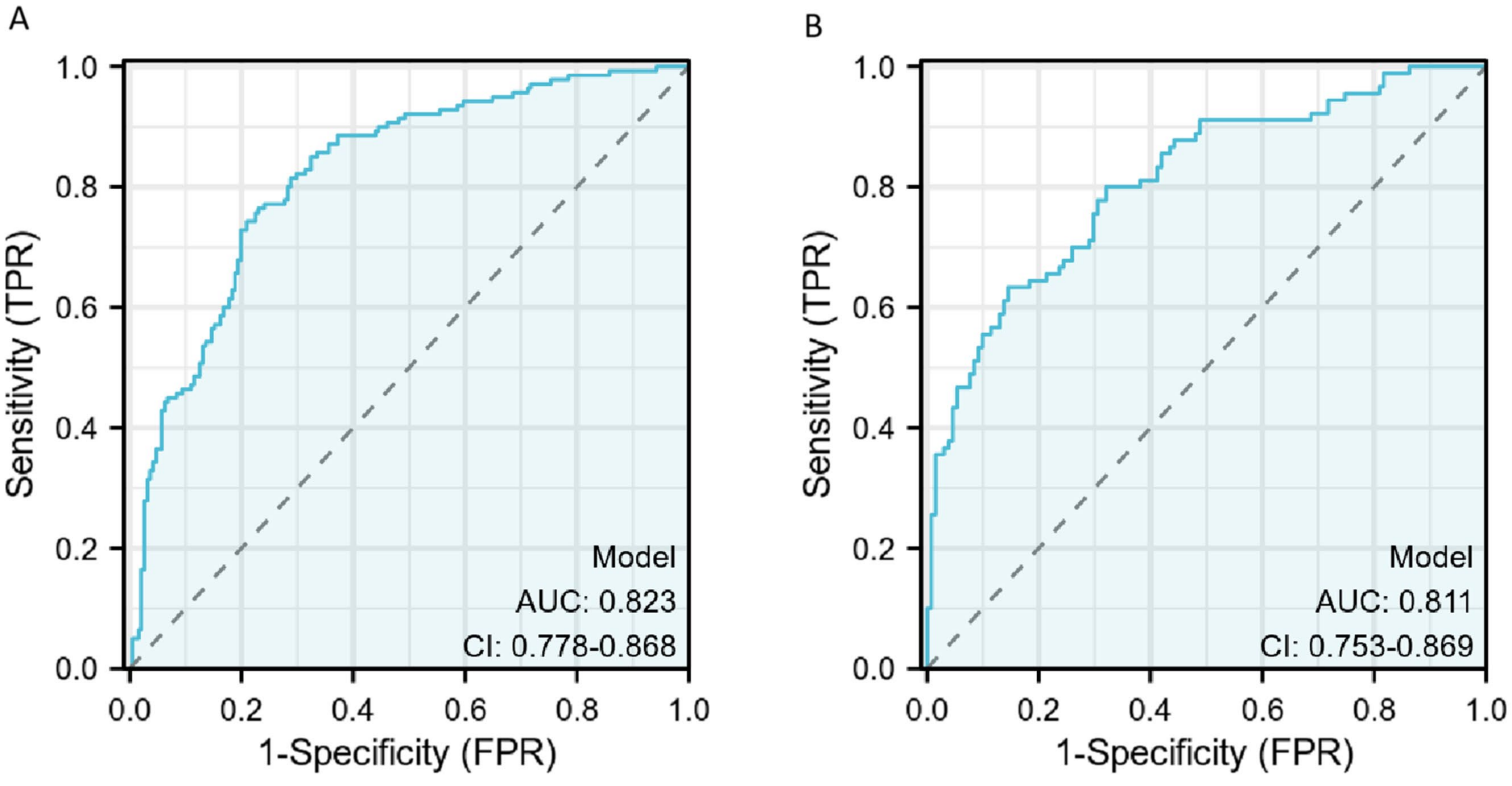
The ROC curve of diagnosis model for training cohort **(A)** and validation cohort **(B)**. AUCs of the nomogram models. AUC, area under the receiver operating characteristic curve.

**Figure 5:**
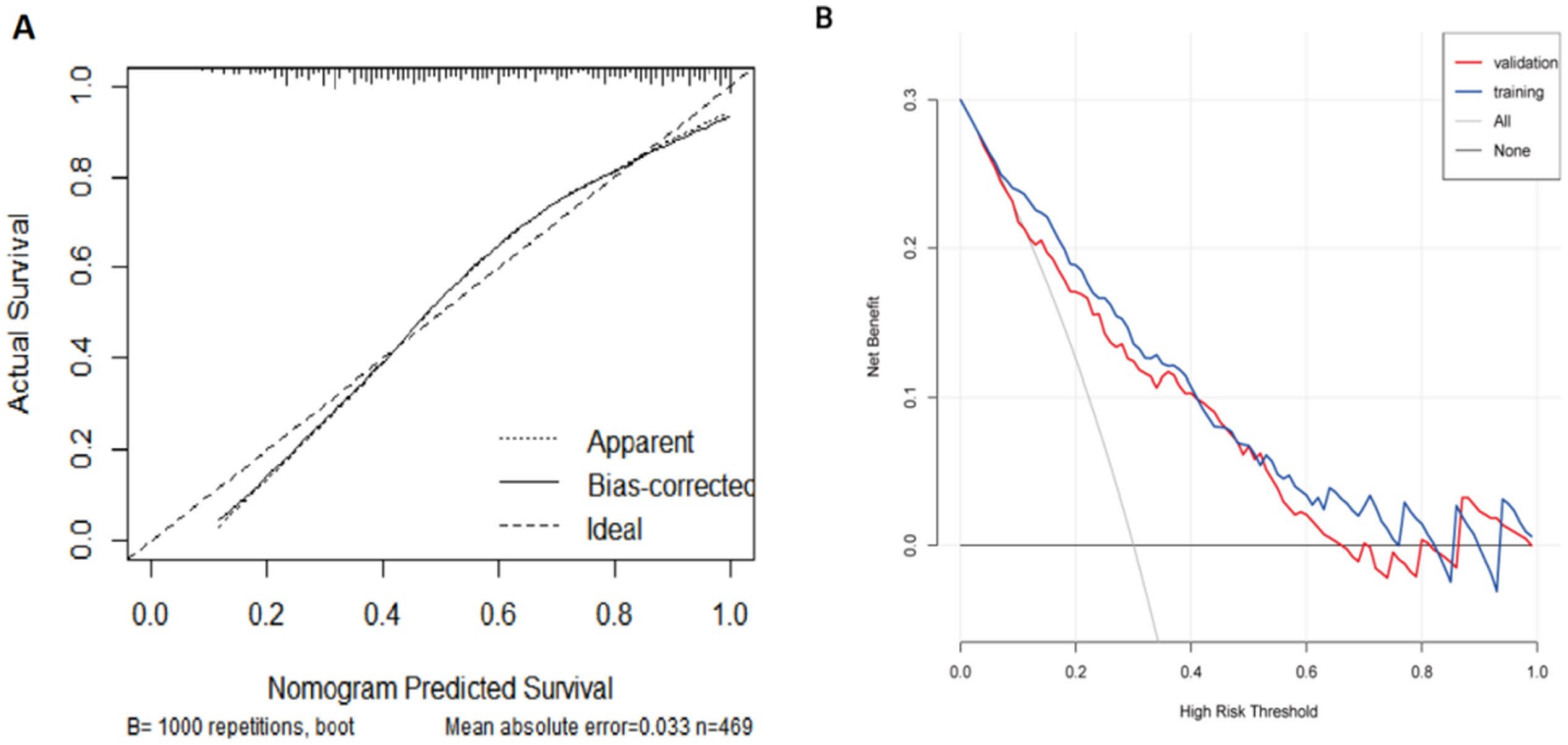
**(A)** A calibration curve of the nomogram in the training set. The dotted line indicates an ideal model, and the solid line indicates the predictive performance of the nomogram. The closer the distance between 2 lines, the better the performance of the nomogram. **(B)** The decision curve analysis of the nomogram for FRI in training cohort and validation cohort. The black line indicates the net benefit when no individuals develop FRI, while the grey line indicates the net benefit when all individuals suffer from FRI. The red line represents in the training cohort, and the blue line represents in the validation cohort. The area among the black line, grey line, red line and blue line indicates the clinical usefulness of the nomogram. FRI, fracture-related infection.

The diagnostic efficacy of the model is summarized in Table 5, and the ROC curve is illustrated in Figure 4. FRID-PI demonstrated 0.823 (95%CI 0.778–0.868) AUC, 77.0% specificity, 76.4% sensitivity, and 76.7% diagnostic accuracy in the training cohort. Validation cohort yielded a 0.811 (95% CI 0.753–0.869) AUC, 85.5% specificity, 63.3% sensitivity, and 76.5% diagnostic accuracy for FRID-PI. The Calibration curve (Figure 5A) demonstrated the degree of consistency between predicted probability and observed probability. The Hosmer–Lemeshow test resulted in a *p*-value of 0.053, suggesting a good fit for the predictive model.

**Table 5:** Model diagnostic efficacy.

	AUC (95% CI)	Sensitivity (%)	Specificity (%)	Accuracy (%)	NPV	PPV	P
Training	0.823 (0.778–0.868)	0.764	0.770	0.767	0.817	0.709	<0.001
Validation	0.811 (0.753–0.869)	0.633	0.855	0.765	0.773	0.75	<0.001

DCA curves in the training and validation cohorts are presented in Figure 5B. Quantifying net benefit probabilities across the 0.0–1.0 threshold, DCA evaluated the clinical utility of FRID-PI. The decision curve indicated a direct correlation between the model’s clinical decisions and net benefit, with greater benefit observed when decisions were farther from the two extreme curves. The current decision curve highlights the high net benefit demonstrated by the FRID-PI for diagnosing FRI.

## Discussion

In this retrospective study, we developed and evaluated a novel diagnostic nomogram based on SUV_max_, SII, IL-6 and ESR to diagnose FRI in patients without clinical confirmatory criteria. The nomogram exhibited high performance and proved beneficial for diagnosing FRI.

Despite advancements in orthopedic surgical techniques, the persistent challenge of post-operative infections remains, exacerbated by the insidious nature of the disease. Additionally, the emergence of culture-negative FRIs, often linked to antibiotic overuse, poses diagnostic challenges. Indeed, swift identification and treatment are crucial to prevent adverse outcomes, necessitating effective and simplified diagnostics.

Originally employed for oncology, ^18^F-FDG PET/CT has gained increasing recognition in inflammatory diseases (12). As early as 1998, scholars explored its utility in diagnosing chronic osteomyelitis (24). However, it should be borne in mind that technological advancements over time limit direct comparisons with historical outcomes due to evolving techniques. Recent studies have increasingly validated the significant role of ^18^F-FDG PET/CT in diagnosing and differentiating osteomyelitis (25–27). Van Vliet et al. (26) conducted a retrospective assessment to evaluate the efficacy and optimal diagnostic accuracy of ^18^F-FDG PET/CT in distinguishing delayed unions in 30 patients. Their findings revealed a significant distinction in SUV_max_ values between aseptic delayed unions and septic delayed unions, yielding an AUC of 0.747. This suggests that SUV_max_ could be a useful differentiation test in bone infection or aseptic pain.

Martina Sollini et al. (28) retrospectively tested the efficacy and optimal diagnostic accuracy of ^18^F-FDG PET/CT in distinguishing infection in non-union in 47 patients. Their results substantiated that ^18^F-FDG PET/CT offers heightened utility when clinical presentations are atypical in suspected FRIs. Notably, the study observed that ^18^F-FDG PET/CT exhibited good performance in ruling out infection among patients with normal CRP levels, contrasting with its efficacy in confirming infection in those with elevated CRP. The authors also found that a combination of visual approach and semi-quantitative analysis achieved a balanced compromise between sensitivity and specificity (80 and 77%), aligning with findings from other notable studies (27). As previously reported (26), a balanced sensitivity and specificity can help avoid unnecessary surgeries and reduce the risk of under treatment in septic delayed unions. Using SUV_max_ for interpreting ^18^F-FDG PET/CT imaging seems promising for FRI, especially in patients without obvious clinical presentations, compensating for the limitations of blood tests.

A complete blood count, coagulation function tests, and routine inflammation indicators (e.g., ESR, CRP) are commonly ordered when FRI is suspected, with blood routine and coagulation indicators being essential prerequisites for pre-operative assessment.

Inflammatory markers (e.g., ESR, WBC, CRP) are recognized as pivotal in clinical guidelines, serving as important suggestive criteria (6). CBC ratio parameters studied more in recent years in infectious diseases (29), were included in our study. Strony et al. (30) used the platelet count to mean platelet volume ratio and reported an AUC of 0.814 for the diagnosis of FRI, outperforming other inflammation indicators. With the in-depth development of research, scholars have found that new indicators composed of multiple types of cells, such as SII and SIRI, can further integrate the combined effects of various inflammatory cells to evaluate the inflammatory level of diseases. Telang et al. (31) research and other studies have shown that patients with elevated SII are prone to developing PJI, and it is an independent risk factor for postoperative infection. These indicators are capable of providing a more comprehensive assessment of the relationship between diseases, immune cells, and the inflammatory response. Therefore, our study also sought to assess the value of the derived indicators based on the existing results.

In this study, we combined ^18^F-FDG PET/CT imaging with laboratory tests to construct a multivariable logistic regression model, including ^18^F-FDG PET/CT semi-quantitative analysis and ESR, SII, IL-6. The model exhibited good discrimination in the training cohort (AUC = 0.823) and the validation cohort (AUC = 0.811). For the training set, the model demonstrated a sensitivity of 76.4%, specificity of 77.0%, and accuracy of 76.7%. There are relatively few diagnostic models for FRI. To assess the clinical utility of the model, we extended our evaluation beyond traditional performance measures like AUC. DCA was employed to estimate the net benefit of the model across a range of risk thresholds, allowing us to evaluate the potential impact of different risk thresholds (32, 33). The research results of Xu et al. (34) on the diagnosis of lower limb FRI using inflammatory indicators showed that the area under the curve (AUC) of the combination of C-reactive protein (CRP) and neutrophil-to-lymphocyte ratio (NLR) could reach 0.873, which is higher than that of the model in this study. However, their research lacks data from a validation group. Therefore, further research is still needed.

Several limitations need to be acknowledged in the current study. Firstly, a notable limitation of our study is the relatively low sensitivity (62.6% in the validation cohort) of the FRID - PI model, risking under diagnosis. FRI’s complexity is a key factor. Our sample size may be too small. A larger one could improve the model’s ability to identify FRI patterns and increase sensitivity. Despite this, the model shows good overall discriminative power. Future research should aim to enhance sensitivity by exploring specific biomarkers or features, using advanced algorithms, and conducting large - scale multicenter studies. Secondly, the analysis was limited to a semi-quantitative assessment of PET/CT data. While visual analysis could potentially offer important diagnostic insights, it is more susceptible to subjective influence. Thirdly, the lack of standardized scan reconstruction resulted in semi-quantitative analysis results specific to the employed camera system, making it challenging to extrapolate findings to other hospitals and camera systems. Given the influence of various factors on SUV_max_ (35), the adoption of standardized criteria such as European Association Research Ltd. (EARL) (36) and Quantitative Imaging Biomarker Alliance (QIBA) (37) for PET/CT scans is recommended. Unfortunately, these reconstruction protocols are not yet integrated into clinical routines in China. And ^18^F-FDG PET/CT is indeed more expensive, and its large-scale clinical application is limited. Fourth, because of lasso regression decreases variability in the parameter estimates and reduces overfitting, leading to fewer parameters in nomogram model. Therefore, expect more machine learning algorithms to be used for diagnosing FRI in the future. Fourth, our study was a single-center, retrospective analysis, underscoring the necessity for multicenter clinical and prospective trials to validate the external applicability of our model. Finally, our study did not follow up with patients, and more attention should be paid in future studies.

## Conclusion

In conclusion, this study makes the first attempt to integrate ^18^F-FDG PET/CT semi-quantitative analysis with laboratory examinations for FRI diagnosis. We developed and validated the FRID-PI, incorporating ESR, SII, IL-6 and SUV_max_ into the diagnostic model. The nomogram, serving as a non-invasive predictive tool, enhances diagnostic accuracy, specificity, and sensitivity of FRI staging when compared to SUV_max_ and ESR, SII, IL-6 alone. This tool holds promise for assisting clinicians in identifying and diagnosing FRI in patients lacking clinical confirmatory criteria, thereby aiding in timely and appropriate treatment decisions.

## Data Availability

The original contributions presented in the study are included in the article material, further inquiries can be directed to the corresponding authors.
